# Association Between Physical Activity Frequency and Depression Severity Among Adults in the United States With Mild to Moderate Depression: A Cross-Sectional Analysis Using National Health and Nutrition Examination Survey (NHANES) Data

**DOI:** 10.7759/cureus.90509

**Published:** 2025-08-19

**Authors:** Oluwabukola C Oyeleye-Adegbite, Oluwanifesimi D Olu-Lawal, Rasheedat A Busari, Joan O Osaigbovo, Cynthia Okoro, Anh N Nguyen, Okelue E Okobi

**Affiliations:** 1 Public Health, Texas A&M University, College Station, USA; 2 Psychiatry and Behavioral Sciences, Jesse Brown Veterans Affairs (VA) Medical Center, Chicago, USA; 3 Medicine, New York City Health and Hospitals Corporation (NYCHHC) Kings County Hospital, Brooklyn, USA; 4 Family Medicine, Nnamdi Azikiwe University, Nnewi, NGA; 5 Family Medicine, Richmond Gabriel University, Arnos Vale, VCT; 6 Hospital Medicine, Luminis Health, Annapolis, USA; 7 Family Medicine, Larkin Community Hospital Palm Springs Campus, Hialeah, USA; 8 Family Medicine, IMG Research Academy and Consulting, Homestead, USA

**Keywords:** depression, mental health, nhanes, phq-9, physical activity, socioeconomic factors

## Abstract

Introduction: Depression is a major contributor to global disease burden. While physical activity is often promoted as a protective factor, its independent association with depression severity remains unclear in population-based samples. This study examined the relationship between moderate-to-vigorous physical activity (MVPA) frequency and depression severity among U.S. adults with mild to moderate depressive symptoms.

Methods: We analyzed data from the 2015-2016 and 2017-2018 cycles of the National Health and Nutrition Examination Survey. Adults aged ≥18 years with Patient Health Questionnaire-9 (PHQ-9) scores between 5 and 14 were included. The outcome was total PHQ-9 score; the exposure was MVPA frequency, categorized as low (one to two days per week) and moderate/high (three or more days per week). Survey-weighted linear regression was used to adjust for demographic and clinical covariates.

Results: Among 1,865 participants (representing 41.2 million U.S. adults), MVPA three or more days per week was not significantly associated with depression severity (β = 0.141, p = 0.48). However, higher income-to-poverty ratio (β = -0.173, p < 0.01) and older age (β = -0.012, p < 0.01) were linked to lower PHQ-9 scores. Racial/ethnic minorities reported significantly higher depression scores than Mexican Americans.

Conclusion: Physical activity frequency was not independently associated with depression severity. Socioeconomic and demographic factors were stronger predictors, highlighting the importance of addressing social determinants in depression interventions.

## Introduction

In the United States, depression is a significant issue in terms of its contribution to functional disability, loss of productivity, and poor quality of life [1]. The National Institute of Mental Health estimated that approximately 21 million adults had at least one significant depressive episode in 2021, and many others had subclinical or minimal to moderate cases [2]. Depression, especially mild or moderate depression, is not always recognized or treated because of the stigma surrounding it, the availability of mental health care, or ignorance [3]. However, even the nonsevere depressive symptoms can severely affect daily functioning, interpersonal interaction, and health condition in case they are not treated [4,5].

One of the growing areas of interest in the management of depression is the role of lifestyle factors, particularly physical activity [6]. This study aims to evaluate the correlation between the frequency of moderate-to-vigorous physical activity (MVPA) and depression severity within the large-scale adult population, while accounting for the sociodemographic and clinical confounders.

Exercise is increasingly being cited not just as a physical good but also for its astounding psychological impacts [7]. It has been observed in multiple clinical trials and observational studies that consistent exercise may help depressed patients restore some level of emotional balance by alleviating depression symptoms, improving moods and overall emotional status [8,9]. Exercise enhances the secretion of endorphins and neurotransmitters like serotonin and dopamine that are usually linked in the neurobiology of depression [10]. Moreover, exercise can contribute to an anti-inflammatory effect, enhanced sleep, and an increased feeling of self-esteem, which are all considered to affect depressive symptoms [11].

Although the present body of evidence grows, the available sources mostly discuss the population of individuals with major depressive disorders (MDDs) or those in treatment, and less clarity is offered regarding the population of individuals with mild to moderate depressive symptoms [12,13]. This category, however, can also be viewed as a critical intervention point since early lifestyle intervention can prove to stop the evolution of depression into a more serious state [14]. Identification of potentially modifiable aspects of behavior, like exercise routines, which are associated with less severe symptoms in this group, is an important potential avenue of research with potential implications on population-level prevention levers and health recommendations [15,16].

The National Health and Nutrition Examination Survey (NHANES), as a nationally representative data source, is a good chance to investigate the relation between physical activity and depression in various settings among people residing in the community [17,18]. NHANES gathers detailed health and lifestyle information (standardized measures of the severity of depression, including the Patient Health Questionnaire-9, PHQ-9, and self-reported frequency of physical activity) [19]. The large-scale trends and associations that can be studied by using this dataset are generalizable to the whole population of U.S. adults.

The study will help to determine the presence of a relationship (hypothesis): physical activity that is more frequent is associated with lower PHQ-9 scores than their less active counterparts. As the nature of the physical activity intervention is easily accessible and inexpensive, identifying an appropriate connection between the frequency of exercise and depressive symptoms may facilitate the process of promoting physical activity as a preventative and treatment tool in the context of depression within the population [20]. The objective of the study is to evaluate the association between weekly exercise frequency and PHQ-9 scores among U.S. adults with mild to moderate depressive symptoms.

## Materials and methods

Study design and data source

This study employed a cross-sectional design using data from the NHANES, a program conducted by the Centers for Disease Control and Prevention (CDC) to assess the health and nutritional status of the civilian, noninstitutionalized U.S. population. NHANES uses a complex, multistage probability sampling design to ensure nationally representative estimates. Data collection includes in-home interviews, standardized physical examinations, and laboratory testing administered at mobile examination centers. For this analysis, publicly available datasets from two consecutive NHANES cycles (2015-2016 and 2017-2018) were used [21]. These cycles were selected because they contain complete information on depression symptoms, physical activity frequency, and relevant covariates.

Study population

The study population comprised adults aged 18 years and older who completed both the PHQ-9 and the physical activity questionnaire. Individuals were included if their PHQ-9 total score ranged from 5 to 14, corresponding to mild (5-9) or moderate (10-14) depressive symptoms. This restriction ensured that the study focused on a population in which lifestyle interventions such as physical activity may have the most clinically significant impact, without the confounding influence of severe depressive pathology. Participants were excluded if they had missing data on key exposure, outcome, or covariate variables, or if they were pregnant at the time of data collection. The final analytic sample was weighted to reflect the U.S. adult population by applying NHANES-specified sampling weights and accounting for stratification and clustering.

Outcome variable: depression severity

Depression severity was assessed using the PHQ-9, a validated nine-item self-report instrument designed to screen for the presence and severity of depressive symptoms. Each item is scored from 0 (not at all) to 3 (nearly every day), generating a total score ranging from 0 to 27. For this analysis, the PHQ-9 total score was used as a continuous outcome variable, providing a linear measure of depression severity. This approach allows for greater statistical power and the ability to detect dose-response relationships between physical activity and depression.

Exposure variable: physical activity frequency

The primary exposure variable was the self-reported frequency of engagement in MVPA over the past seven days. NHANES assesses physical activity through two items: the number of days participants performed vigorous-intensity recreational activities (e.g., running and aerobics) and the number of days they engaged in moderate-intensity activities (e.g., brisk walking and gardening). These were derived from the variables PAQ605 (vigorous days) and PAQ620 (moderate days). A composite variable was created by summing the total number of moderate and vigorous activity days per week, capped at seven. For analysis, this variable was categorized into two groups: low frequency (one to two days) and moderate/high frequency (three or more days per week). This categorical exposure variable was used to facilitate the interpretation of results and identify potential thresholds of activity associated with reduced depressive symptoms.

Covariates and confounders

Several sociodemographic, behavioral, and health-related variables known to be associated with both physical activity and depression were included as covariates to minimize confounding. These included age (continuous), sex (male/female), race/ethnicity (non-Hispanic White, non-Hispanic Black, Hispanic, other), and income-to-poverty ratio (continuous). Behavioral covariates included smoking status (yes/no). Clinical variables included body mass index (BMI; continuous), presence of hypertension, diabetes, and history of cardiovascular disease. All variables were selected a priori based on existing literature and theoretical relevance using a conceptual framework grounded in the biopsychosocial model of mental health.

Statistical analysis

All analyses were conducted using Stata version 18.0 (StataCorp, College Station, TX), incorporating the complex survey design of NHANES using the svy suite of commands. Survey weights (wtint2yr), strata (sdmvstra), and primary sampling units (PSUs; sdmvpsu) were applied to ensure nationally representative estimates. As recommended by the National Center for Health Statistics (NCHS), sample weights for each two-year cycle were divided by the number of cycles used (n = 2) to obtain accurate nationally representative estimates across the four-year combined dataset (2015-2018).

Descriptive statistics were first used to summarize the distribution of demographic and clinical characteristics across physical activity frequency categories. Means and standard deviations were reported for continuous variables, while categorical variables were summarized using frequencies and percentages. In the bivariate analysis phase, unadjusted relationships between physical activity frequency and depression scores, as well as other covariates, were examined using survey-adjusted statistical tests. For categorical variables, design-based F-statistics were employed rather than standard chi-square tests, as required by the NHANES complex sampling design. These F-statistics are derived from the Pearson chi-square values but are adjusted to reflect the effects of sampling weights, clustering, and stratification, thereby providing valid inference for survey data. Whereas for continuous variables, a survey-weighted t-test was used to assess mean differences.

Multivariable survey weight linear regression models were then fitted to assess the association between physical activity frequency and PHQ-9 scores. All models were adjusted for the covariates listed above.

Regarding missing data, participants with incomplete information on any variable included in the regression model were excluded using complete-case (list-wise) analysis. Although multiple imputation is a common strategy to handle missing data, it was not applied in this study due to the structural complexity of the NHANES design. Specifically, incorporating imputation methods while preserving the survey’s multistage sampling features, such as weights, strata, and PSUs, is statistically intricate and not readily compatible with standard imputation algorithms in most software environments. Additionally, the overall proportion of missingness across key variables was relatively low, and the final sample size remained sufficiently large to maintain statistical power. Therefore, complete-case analysis was deemed both methodologically sound and practically appropriate to maintain the fidelity of nationally representative estimates.

Ethical considerations

This study used deidentified, publicly available data from NHANES, which is conducted by the CDC and approved by the NCHS Research Ethics Review Board. All participants in NHANES provide written informed consent at the time of data collection. Because the current analysis involved secondary data with no identifiable information, additional Institutional Review Board approval was not required under U.S. federal regulations: 45 Code of Federal Regulations 46.104(d)(4). Nonetheless, the study adhered to ethical principles of human subjects research, including respect for persons, beneficence, and justice.

## Results

Table 1 presents the weighted sociodemographic and clinical characteristics of U.S. adults with mild to moderate depressive symptoms, stratified by physical activity frequency into two groups: those engaging in low MVPA (one to two days per week) and those with moderate/high MVPA (three or more days per week). The analysis reveals several statistically significant differences between the groups.

**Table 1:** Survey-weighted sociodemographic and clinical characteristics of U.S. adults with mild to moderate depression, by physical activity frequency Data are weighted using NHANES sample weights and represent the U.S. noninstitutionalized adult population with mild to moderate depressive symptoms (PHQ-9 score 5-14). Categorical data are reported as numbers and weighted percentages. Continuous variables are presented as mean ± standard deviation. T-tests and design-based F-tests were used to assess differences between the physical activity groups Statistical significance: *p < 0.05, **p < 0.001 MVPA: moderate-to-vigorous physical activity; CI: confidence interval; NHANES: National Health and Nutrition Examination Survey; PHQ-9: Patient Health Questionnaire-9

Variable	Low (1-2 days) MVPA (n = 9,785,565)	Moderate/high (≥3 days) MVPA (n = 31,411,917)	T/F-test	p value
Age in years at screening	43.41 ± 14.83 (95% CI: 43.32-43.50)	48.93 ± 17.77 (95% CI: 48.87-48.99)	T = -3.67	0.001^**^
Body mass index (kg/m^2^)	29.92 ± 7.24 (95% CI: 29.88-29.96)	30.68 ± 7.74 (95% CI: 30.65-30.71)	T = -1.25	0.22
Depression severity	7.63 ± 2.35 (95% CI: 7.62-7.64)	7.72 ± 2.60 (95% CI: 7.71-7.73)	T = -0.51	0.63
Ratio of family income to poverty	2.47 ± 1.48 (95% CI: 2.46-2.48)	2.58 ± 1.68 (95% CI: 2.57-2.59)	T = -1.08	0.29
Gender				
Male	5,226,584 (32%)	11,215,174 (68%)	F = 12.90	0.001^**^
Female	4,558,981 (18%)	20,196,742 (82%)		
Smoked at least 100 cigarettes in life				
Yes	6,384,451 (29%)	15,972,778 (71%)	F = 13.08	0.001^**^
No	3,401,114 (18%)	15,439,138 (82%)		
Ever told you had high blood pressure				
Yes	3,235,166 (20%)	12,994,605 (80%)	F = 3.54	0.07
No	6,550,399 (26%)	18,417,312 (74%)		
The doctor told you have diabetes				
Yes	958,199 (16%)	5,084,839 (84%)	F = 5.54	0.03^*^
No	8,827,366 (25%)	26,327,078 (75%)		
Ever told you had a stroke				
Yes	362,625 (19%)	1,546,326 (81%)	F = 0.74	0.40
No	9,422,940 (24%)	29,865,590 (76%)		
Race/ethnicity				
Mexican American	742,950 (22%)	2,682,834 (78%)	F = 1.16	0.33
Other Hispanic	664,470 (25%)	2,046,659 (75%)		
Non-Hispanic White	6,650,637 (25%)	20,186,632 (75%)		
Non-Hispanic Black	806,865 (18%)	3,696,534 (82%)		
Non-Hispanic Asian	920,641 (25%)	2,799,256 (75%)		
Other race	9,785,565 (24%)	31,411,917 (76%)		

Participants in the moderate/high MVPA group were significantly older than those in the low MVPA group (48.93 ± 17.77 vs. 43.41 ± 14.83 years, t = -3.67, p = 0.001), suggesting that older adults in this sample were more likely to engage in regular physical activity. However, no statistically significant difference was observed in BMI, with similar mean BMI values reported across groups (30.68 ± 7.74 vs. 29.92 ± 7.24; p = 0.22).

Depression severity, measured by the PHQ-9 total score, did not differ significantly between the two physical activity groups (7.72 ± 2.60 vs. 7.63 ± 2.35; p = 0.63), indicating that, at the unadjusted level, depression symptoms were comparable regardless of MVPA frequency. Similarly, the ratio of family income to poverty was slightly higher in the moderate/high MVPA group (2.58 ± 1.68) than in the low MVPA group (2.47 ± 1.48), but this difference was not statistically significant (p = 0.29).

Significant gender differences were observed across MVPA categories (F = 12.90, p = 0.001). Among those with low MVPA, 5,226,584 (32%) were male and 4,558,981 (18%) were female, whereas in the moderate/high MVPA group, the majority were female 20,196,742 (82%) compared to 11,215,174 (68%) males. This suggests a possible gender disparity in physical activity levels among individuals with depressive symptoms.

Smoking status was also significantly associated with MVPA frequency (F = 13.08, p = 0.001). A higher proportion of participants who had smoked at least 100 cigarettes in their lifetime were in the moderate/high MVPA group, 15,972,778 (71%), compared to the low MVPA group, 6,384,451 (29%). This may reflect lifestyle clustering, where individuals who are active might also engage in other risk or compensatory behaviors.

Hypertension and diabetes showed mixed associations. Although the difference in hypertension prevalence approached statistical significance (F = 3.54, p = 0.07), diabetes was significantly less common among individuals with low MVPA 958,199 (16%) compared to those in the moderate/high MVPA group (5,084,839 or 84%; F = 5.54, p = 0.03). Stroke prevalence did not differ significantly between groups (p = 0.40). Notably, statistically significant association (F = 5.54, p = 0.03) was found between diabetes and MVPA levels, with individuals in the low MVPA group (one to two days per week) having a significantly lower prevalence of diabetes (16%) than the moderate/high MVPA group (three or more days per week; 84%). Lastly, no statistically significant association was found between race/ethnicity and physical activity level (F = 1.16, p = 0.33). Across all racial/ethnic subgroups, the majority of participants engaged in moderate/high MVPA, ranging from 2,046,659 (75%) for other Hispanic to 3,696,534 (82%) in Non-Hispanic black.

Multivariate weighted regression

Table 2 presents the results of a survey-weighted multivariable linear regression model evaluating the association between MVPA frequency and depression severity among U.S. adults with mild to moderate depressive symptoms (PHQ-9 scores 5-14). The model adjusts for key sociodemographic and clinical covariates, including age, gender, income-to-poverty ratio, smoking status, BMI, history of hypertension, diabetes, stroke, and race/ethnicity. Depression severity was modeled as a continuous outcome, and physical activity was categorized into low (one to two days per week) and moderate/high (three or more days per week) MVPA groups. All analyses accounted for the complex NHANES survey design using appropriate sampling weights, strata, and PSUs. Regression coefficients (β), standard errors, and statistical significance levels are reported. This model aims to determine whether a higher frequency of physical activity is independently associated with lower depression severity after adjusting for potential confounding factors.

**Table 2:** Survey-weighted multivariable linear regression assessing the association between physical activity and depression severity among U.S. adults with mild to moderate depression Depression severity was measured using the PHQ-9 total score as a continuous outcome variable. The primary exposure was MVPA, categorized as low (one to two days per week, reference) vs. moderate/high (three or more days per week). All models were survey-weighted using NHANES sampling weights and adjusted for sociodemographic and clinical covariates. Standard errors are in parentheses Significance thresholds: *p < 0.05, **p < 0.01, ***p < 0.001 MVPA: moderate-to-vigorous physical activity; PHQ-9: Patient Health Questionnaire-9; NHANES: National Health and Nutrition Examination Survey

Depression severity	Adjusted model (n = 1,865)
MVPA categories	
Moderate/high (≥3 days)	0.141 (0.209)
Age in years at screening	-0.012(0.003)^**^
Gender	
Female	0.292 (0.155)
Ratio of family income to poverty	-0.173 (0.052)^**^
Smoked at least 100 cigarettes in life	
No	-0.365 (0.191)
Body mass index (kg/m^2^)	-0.006 (0.009)
Ever told you had high blood pressure	
No	-0.355 (0.186)
Doctor told you have diabetes	
No	-0.247 (0.279)
Ever told you had a stroke	
No	-0.159 (0.283)
Race/ethnicity	
Other Hispanic	0.622 (0.210)^**^
Non-Hispanic White	0.488 (0.285)
Non-Hispanic Black	0.619 (0.229)^* ^
Non-Hispanic Asian	0.900 (0.315)^**^
Constant	8.867 (0.478)^***^
R^2^	0.036

**Figure 1:**
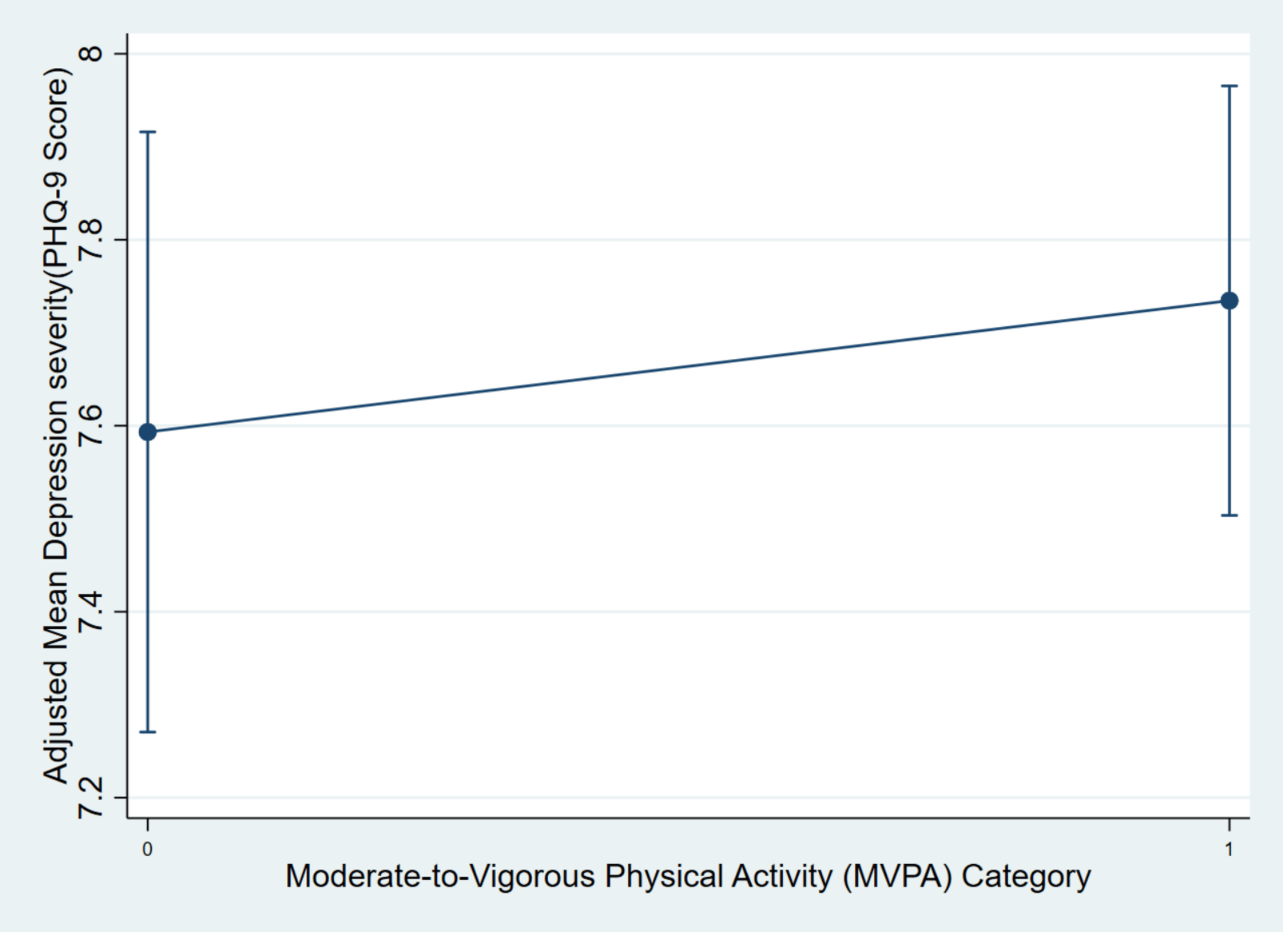
Adjusted mean depression severity (PHQ-9 scores) by MVPA frequency among U.S. adults with mild to moderate depression The adjusted mean PHQ-9 scores with 95% confidence intervals by MVPA category (0 = low activity, one to two days per week; 1 = moderate/high activity, greater than or equal to three days per week). Results are derived from the fully adjusted survey-weighted linear regression model in Table 2, which controls for age, gender, income-to-poverty ratio, smoking, BMI, hypertension, diabetes, stroke, and race/ethnicity. Analyses account for NHANES survey design features using appropriate weights, strata, and primary sampling units MVPA: moderate-to-vigorous physical activity; PHQ-9: Patient Health Questionnaire-9

In the fully adjusted survey-weighted linear regression model, moderate-to-high physical activity frequency (three or more days per week) was not significantly associated with lower depression severity compared to low activity frequency (one to two days per week). The coefficient for the MVPA variable was positive (β = 0.141), but this result was not statistically significant (p > 0.05), suggesting that after accounting for covariates, there was no clear evidence of an independent association between higher physical activity and lower depressive symptoms in this population. Figure 1 further illustrates this finding, showing overlapping confidence intervals and a slight, non-significant increase in adjusted mean PHQ-9 scores among those engaging in higher MVPA levels. This visual alignment with the regression model underscores the null association between physical activity frequency and depression severity in the study sample.

Age was significantly associated with depression severity, with each additional year of age associated with a slight but significant reduction in PHQ-9 score (β = -0.012, p < 0.01). This finding indicates that younger individuals with mild to moderate depression may report higher depressive symptom burden compared to older adults, after adjusting for other variables.

The ratio of family income to poverty was inversely associated with depression severity (β = -0.173, p < 0.01), indicating that participants with higher income relative to the poverty threshold reported significantly lower depression scores. This aligns with existing literature demonstrating socioeconomic disadvantage as a risk factor for greater depressive symptomatology.

Gender was not significantly associated with depression severity. While females showed a higher average PHQ-9 score compared to males (β = 0.292), the association did not reach statistical significance (p > 0.05).

Smoking status, BMI, and diagnoses of hypertension, diabetes, or stroke were not significantly associated with PHQ-9 scores in the adjusted model. For instance, having never smoked was associated with a lower PHQ-9 score (β = -0.365), but the association was not statistically significant (p > 0.05). Similarly, the presence or absence of chronic conditions such as diabetes, hypertension, and stroke did not significantly impact depression severity, suggesting that these conditions may not independently explain variability in depressive symptoms once other covariates are considered.

Race/ethnicity showed several statistically significant associations with depression severity. Compared to the reference group (Mexican Americans), participants identifying as Other Hispanic (β = 0.622, p < 0.01), Non-Hispanic Black (β = 0.619, p < 0.05), and Non-Hispanic Asian (β = 0.900, p < 0.01) all reported significantly higher PHQ-9 scores. These findings suggest potential racial/ethnic disparities in depression symptom burden among adults with mild to moderate depressive symptoms, which may be influenced by structural, cultural, or access-related factors.

Figure 1 displays the adjusted mean PHQ-9 depression scores across two categories of self-reported MVPA: low frequency (one to two days per week) and moderate-to-high frequency (three or more days per week). This visual representation was designed to assess the potential dose-response pattern between physical activity frequency and depression severity after adjusting for key sociodemographic and clinical covariates.

## Discussion

This cross-sectional study examined the association between MVPA frequency and depression severity among U.S. adults with mild to moderate depressive symptoms using nationally representative NHANES data from 2015-2018. After adjusting for multiple sociodemographic and clinical covariates, we found that engaging in MVPA on three or more days per week was not independently associated with lower PHQ-9 depression scores. Instead, socioeconomic disadvantage, younger age, and specific racial and ethnic categories emerged as more consistent predictors of depression severity.

The absence of a significant association between MVPA frequency and depression severity in our adjusted models may be partially explained by how physical activity was measured. In our analysis, MVPA was derived from self-reported weekly frequency and did not distinguish between physical activity domains (e.g., leisure-time, occupational, or transport-related activity). Prior research has demonstrated that the mental health benefits of physical activity may vary depending on its domain. Rutherford et al. [22], using NHANES 2011-2014 data, reported that leisure-time MVPA was significantly associated with reduced depression severity, whereas work-related and travel-related activities showed no such association. In their study, U.S. adults who engaged in ≥150 minutes of leisure MVPA per week experienced significantly lower PHQ-9 scores, underscoring the importance of context and purpose in understanding physical activity’s mental health benefits. Our reliance on general MVPA frequency measures without such domain-specific information may therefore have attenuated the observed association.

Socioeconomic status, measured via the income-to-poverty ratio, was significantly and inversely associated with depression severity. Individuals with lower income relative to the federal poverty threshold reported higher PHQ-9 scores, supporting a well-documented link between economic disadvantage and mental health burden [14-24]. Financial insecurity may contribute to psychological stress through reduced access to care, limited coping resources, and increased daily life strain.

Age was also negatively associated with depression severity, indicating that younger adults were more likely to report higher PHQ-9 scores. This finding is consistent with broader epidemiological patterns, where depression tends to peak in early to mid-adulthood, often attributed to career, educational, or familial pressures, and gradually declines with age.

Racial and ethnic disparities were evident in the findings, with participants identifying as Other Hispanic, Non-Hispanic Black, and Non-Hispanic Asian, showing significantly higher depression scores compared to Mexican Americans. These disparities may reflect longstanding inequities in access to mental health care, experiences of discrimination, and differing cultural perceptions of mental health and help-seeking.

Other covariates, such as gender, smoking status, BMI, hypertension, diabetes, and stroke, did not significantly predict depression severity in our adjusted analysis. While these variables are commonly associated with depression risk, their influence may be diminished when controlling for more proximal sociodemographic and behavioral determinants such as age and socioeconomic status.

Choi et al. reported no statistically significant association between self-reported physical activity levels and depressive symptoms in their study population, suggesting that the relationship between exercise and mental health outcomes may be more complex than previously assumed [23]. However, other research has demonstrated promising results regarding the antidepressant effects of physical activity. For instance, one study found that aerobic exercise produced a therapeutic response in individuals with MDD that was comparable to the effects of selective serotonin reuptake inhibitors, a commonly prescribed class of antidepressants [24]. Furthermore, a 16-week clinical trial involving older adults (aged 50 and above) diagnosed with MDD showed that structured exercise interventions were equally effective as pharmacologic treatment in alleviating depressive symptoms [25]. These findings underscore the potential of physical activity as a viable and nonpharmacological alternative or complement to standard antidepressant therapy, particularly in older populations.

Our study showed that more women showed a higher average PHQ-9 score compared to men (β = 0.292), which correlates with similar findings like studies by Huang and Huang, on the association between increased vigorous exercise and decreased depressive symptoms in United States adults using NHANES 2017-2020, where 63% of women had a PHQ-9 score >10, with only 3% of men having this score [3], and a study by Meng et al. on the same subject; there were more women with depression compared to men (63.57% vs. 36.43%) [26]. These results were statistically significant, unlike what was observed in our study. Huang and Huang found a 9% decreased odds for depression, which persisted after controlling for a confounder like gender [3], which differs from our study, where physical activity did not independently affect depression severity. Meng et al. [26] found a more obvious impact of physical activity on depression in men than women, which was similar to a study by Yang et al. [27], where a possible correlation was seen between higher proportions of MVPA and lower risk for depression in men and those 45 years or older. Unlike our study, Meng et al. observed that more physical activity (except walking or bicycling) was significantly and independently associated with a lower risk for depression [26].

Limitations of the study

This study's cross-sectional design limits causal interpretation between physical activity and depression severity. MVPA was self-reported, which may introduce recall and social desirability bias and reduce measurement accuracy. The small number of participants reporting five or more MVPA days per week also limited statistical power.

Important behavioral and clinical confounders such as alcohol use, sleep duration, and education level were excluded due to over 90% missingness. Similarly, data on antidepressant use, anxiety, and comorbidities were either unavailable or incomplete, potentially leading to residual confounding. Although complete case analysis preserved survey design integrity, it assumes data were missing completely at random and may reduce generalizability.

Future studies should use longitudinal or linked NHANES data to assess causality and integrate objective physical activity measures. Inclusion of behavioral and psychosocial factors like alcohol use, sleep, and education, along with advanced missing data strategies, such as multiple imputation, could improve validity and model accuracy.

## Conclusions

In a nationally representative sample of U.S. adults with mild to moderate depressive symptoms, moderate-to-high MVPA (≥3 days/week) was not independently associated with depression severity after adjusting for covariates. Instead, younger age, lower socioeconomic status, and racial/ethnic minority status were significant predictors of higher depressive symptoms. Interventions aimed at reducing depression should address not only physical activity, but also social and structural determinants of mental health.
